# FDTD analysis of a noninvasive hyperthermia system for brain tumors

**DOI:** 10.1186/1475-925X-11-47

**Published:** 2012-08-14

**Authors:** Sulafa M Yacoob, Noha S Hassan

**Affiliations:** 1 Biomedical Engineering Department, Faculty of Engineering, Cairo University, Giza, 12613, Egypt

**Keywords:** Bioheat equation, Specific absorption rate (SAR), Computational modeling, Patch antenna, Ellipsoidal chamber

## Abstract

**Background:**

Hyperthermia is considered one of the new therapeutic modalities for cancer treatment and is based on the difference in thermal sensitivity between healthy tissues and tumors. During hyperthermia treatment, the temperature of the tumor is raised to 40–45°C for a definite period resulting in the destruction of cancer cells. This paper investigates design, modeling and simulation of a new non-invasive hyperthermia applicator system capable of effectively heating deep seated as well as superficial brain tumors using inexpensive, simple, and easy to fabricate components without harming surrounding healthy brain tissues.

**Methods:**

The proposed hyperthermia applicator system is composed of an air filled partial half ellipsoidal chamber, a patch antenna, and a head model with an embedded tumor at an arbitrary location. The irradiating antenna is placed at one of the foci of the hyperthermia chamber while the center of the brain tumor is placed at the other focus. The finite difference time domain (FDTD) method is used to compute both the SAR patterns and the temperature distribution in three different head models due to two different patch antennas at a frequency of 915 MHz.

**Results:**

The obtained results suggest that by using the proposed noninvasive hyperthermia system it is feasible to achieve sufficient and focused energy deposition and temperature rise to therapeutic values in deep seated as well as superficial brain tumors without harming surrounding healthy tissue.

**Conclusions:**

The proposed noninvasive hyperthermia system proved suitable for raising the temperature in tumors embedded in the brain to therapeutic values by carefully selecting the systems components. The operator of the system only needs to place the center of the brain tumor at a pre-specified location and excite the antenna at a single frequency of 915 MHz. Our study may provide a basis for a clinical applicator prototype capable of heating brain tumors.

## Background

Hyperthermia or thermotherapy has often been used alone to induce cancer cell death by elevating the temperature in these cells or as an adjunctive cancer treatment modality to improve the clinical outcome of radiotherapy and chemotherapy [1,2]. During hyperthermia therapy, the tumor is heated to a temperature in the range of 40–45°C for a defined period of time (30–60 min) resulting in damaging cancer cells, while keeping healthy tissue at safe temperatures.

Research interest in the applicability of clinical hyperthermia in cancer therapies is continuously growing. Local, regional, and whole-body hyperthermia treatment regimens have been under extensive investigation during the past decades and have provided promising results with several types of cancers [3-6]. The quality and the effectiveness of hyperthermia treatment is highly dependent on the ability to deposit power and thus, to localize the temperature distribution in the tumor region without harming the surrounding normal tissues. While, hyperthermia has proven to be an effective tool in the treatment of superficial tumors [7,8], further research is still required to sufficiently heat deep seated tumors. In particular, invasive interstitial microwave hyperthermia has been employed successfully for the treatment of tumors within the brain [9-12], but the major problem with this technique is that it requires transcranial implantation into the tumor. Thus, research should be focused on raising the quality of hyperthermia devices used for effective and localized cancer treatment of deeply seated brain tumors.

Several computational studies have proposed non-invasive hyperthermia applicators to heat superficial tumors [13,14]. Specifically, antenna arrays have been used for the application of hyperthermia to superficial regions of the head and neck [15,16]. These applicators are not suitable for effectively heating deep seated brain tumors. Focusing electromagnetic power into tumors deeply located in the brain possesses a challenge since high water content tissues such as blood and muscle absorb this power and rapidly attenuate wave propagation, thus preventing deep penetration into the brain. Therefore, many research attempts were undertaken to develop applicators capable of noninvasively depositing electromagnetic energy into brain tissues without affecting surrounding healthy tissues.

Theoretical studies [17] and laboratory measurements [18,19] were carried out to examine the possibility of using arrays of multiple antennas for heating deeper regions in a neck-mimicking cylinder as well as in phantom models [20-23]. Paulides et al., [23] developed a hyperthermia applicator using an array of multiple dipole antennas. The specific absorption rate (SAR) was computed in a 3D model of the neck containing a lymph node tumor which served as the basis for their prototype. Ishihara et al. [21] designed a reentrant cavity hyperthermia applicator to heat head and neck tumors using a homogeneous tissue phantom model to compute the temperature distribution in their model.

In addition, extensive theoretical and experimental research has been conducted to develop a noninvasive focused hyperthermia system based on the use of a complete ellipsoidal beamformer [24,25]. In these studies, deep and superficial focused hyperthermia was only achieved using a combination of operation frequencies and several dielectric matching layers placed around a spherical head, as well as a 13 tissue head model. Several hot spots were also observed in phantom temperature measurements [26]. Furthermore, a dipole/double discone antenna was used to deposit energy into their head and phantom models. Dipoles, however, require an RF-matching network containing a balun which absorbs a substantial amount of power and thus decreases the efficiency of the antenna used in their prototype. The temperature distribution in brain tumors located at various positions in simulated head models was not investigated in these studies. Zastrow et al. [27] proposed and evaluated the performance of a noninvasive time-multiplexing microwave beamforming technique for selective localized heating of target locations in a numerical head phantom. The microwave source consisted of an array of 134 small antennas placed around the head while deionized water was used as a coupling medium in contact with the surface of the head. While the efficacy of the proposed system was demonstrated, simplification and further optimization of the setup is essential for its application to a clinical hyperthermia system.

Careful selection, design and tuning of the antenna used as a source of tissue irradiation in noninvasive hyperthermia applicators is necessary since the presence of the head comprised of lossy soft tissues in the vicinity of the antenna has a considerable effect on the characteristics and the performance of the antenna.

In addition, the hyperthermia applicator system should provide efficient power absorption distribution (SAR) and selective heating efficacy in deep seated brain tumors. The SAR is a measure of the rate at which electromagnetic energy is absorbed by brain tissues and is a vital quantity in assessing the effectiveness of hyperthermia. The temperature in brain tissues is usually computed by substituting the SAR values into the Pennes bioheat equation. Knowledge of the temperature distribution in brain tissues is essential since the goal of hyperthermia is to raise the temperature of the tumor without affecting healthy brain tissues.

To our knowledge, adequate localization and focusing of energy patterns and the calculation of the temperature distribution in a realistic 3D head model containing a deeply seated as well as a superficial brain tumor due to irradiation by a simple easy to fabricate antenna excited at a single frequency placed in a partial half ellipsoidal noninvasive air filled hyperthermia applicator is still lacking. In this study, we numerically designed a noninvasive hyperthermia applicator system composed of a patch antenna, a partial half ellipsoidal chamber and a head model containing a tumor. The FDTD method was used to compute both the SAR patterns and the temperature distribution in three different head models. Several improvement steps were performed on all of the applicator system configurations to adequately ensure sufficient and focused energy deposition and temperature distribution in the brain tumor. The system is capable of heating deep seated as well as superficial brain tumors by placing the center of the tumor at a pre-specified location (one of the foci) controlled by the operator of the proposed hyperthermia system. The use of a partial half ellipsoidal chamber ensures better head positioning and patients comfort compared to whole ellipsoidals.

## Methods

### Hyperthermia system model

**Figure 1:**
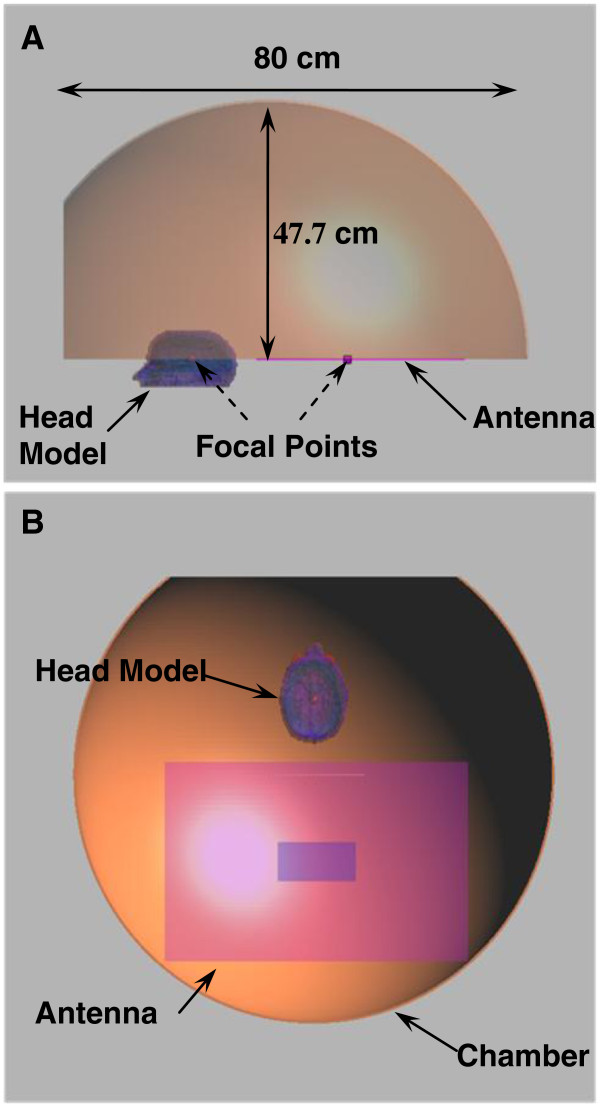
**Hyperthermia chamber model (A) sagittal view, (B) axial view.** The tumor in the head model is placed at one of the foci while the antenna is placed at the other focus.

The proposed focused deep brain hyperthermia model consists of a half ellipsoidal chamber with part of the bottom horizontal walls, coinciding with the major axis, partially covered (60 cm). A vertical cut is removed from the opposite side of the half ellipsoidal chamber leaving room for the head to be placed in the open uncovered part (Figure 1A). The radiating device (antenna) is placed at one of the chambers focal points while the target (brain tumor) is placed at the other focal point. The exact placement of the antenna inside the chamber is depicted in Figure 1B. The distance between the brain tumor placed at one of the focal points of the hyperthermia chamber model and the antenna placed at the other focal point was 30 cm.

This chamber was based on the interesting focusing properties of ellipsoidal reflectors, where if a light source is placed at one focus of the ellipse; all light rays on the plane of the ellipse are reflected to the second focus with the same path length. The walls of the chamber are modeled as a thin shell of copper (5 mm in thickness) having a conductivity of σ = 5.8 × 10^7^ S/m. The system arrangement ensures better directivity, higher energy deposition and better focusing in the brain tumor.

### Chamber model

Different chamber model sizes were simulated to ensure better focusing properties in the brain tumor. The final chamber size was selected based on the design that ensures a better localization and maximum SAR deposition at the target region (tumor) in the head. The final model used in this study had a chamber major axis of 100 cm while the distance from the center of the major axis to the top of the chamber was 47.7 cm. Several vertical sections were deducted from the edge of the chamber close to the second focal point where the brain tumor is placed. A vertical section 20 cm to the right of the second focal point was selected where simulations have shown that the focusing properties into the tumor did not improve when sections were made at larger distances.

### Human head models

**Figure 2:**
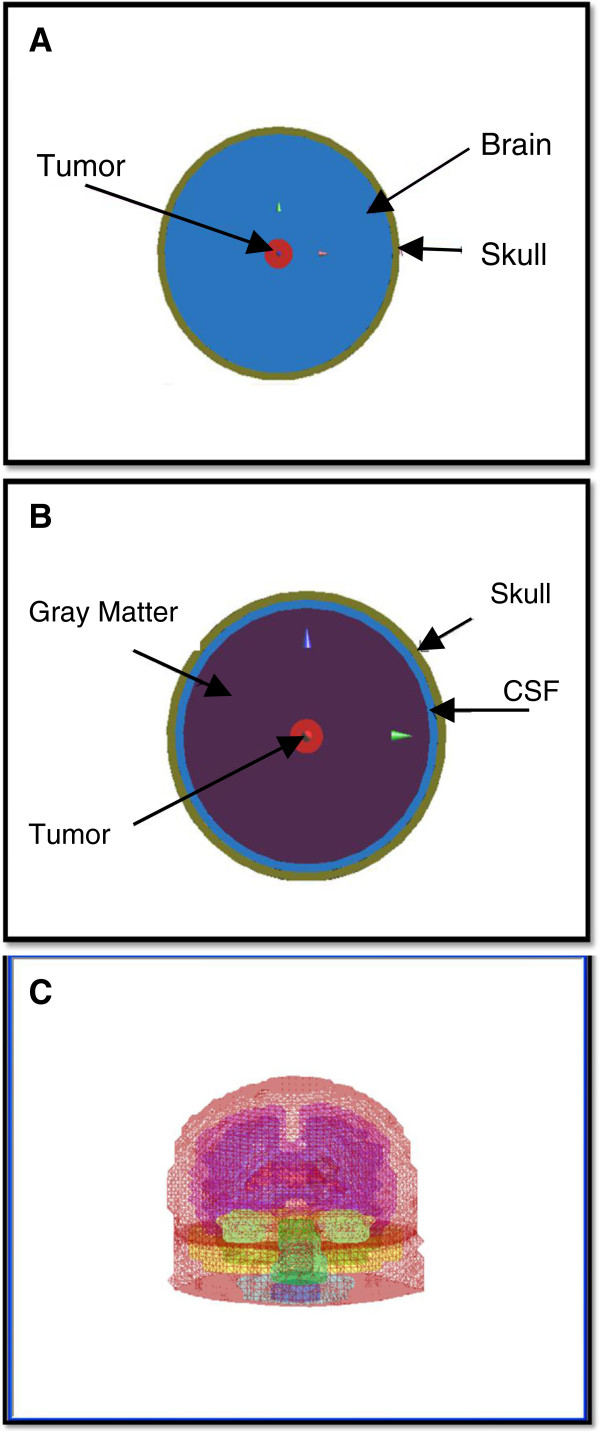
Head Models (A) average brain model, (B) layered concentric sphere model of the brain and (C) realistic 3D head model.

Three head models were used in our computations. First, the average brain model consisted of a two layered sphere representing the simplest head configuration model [2]. The inner sphere representing average brain tissue had a radius of 8.5 cm, while the outer sphere representing the skull had a radius of 9 cm as shown in Figure 2A. The simple head model forms a reference configuration and is farther extended to a more complicated layered concentric spheres head model consisting of three concentric spheres representing the skull, the cerebral spinal fluid (CSF) and the gray matter (GM). Sphere radii were 9 cm, 8.5 cm and 8 cm respectively (Figure 2B). A spherical tumor (20 mm in diameter) was placed at the center of these two head models. Finally, a more complex realistic 3D head model was constructed and used in the final hyperthermia system model. The final 3D realistic head model was constructed to simulate the human head as shown in Figure 2C. A hundred and twenty transverse magnetic resonance imaging (MRI) anatomical slices of a 35 year old male with a brain tumor were segmented, stacked and surface rendered to generate the head model. MR images were acquired by the national cancer institute with patient consent obtained. The head model consisted of seventeen different structures: the skin, skull, CSF, GM, white matter (WM), ventricles, pons, medulla, cerebellum, muscle, fat, mouth cavity, tongue, sinus, eyes, cartilages and a tumor. The spatial resolution of the human head was 2 mm. The size of the brain tumor was approximately 2 cm in diameter and was either centrally located close to the ventricles or superficially placed in the head model. The electrical properties (dielectric constant and conductivity) and the thermal properties used in our computations for different human head tissues were assigned according to the values reported in the literature [28] and the SEMCAD material database. Table 1 lists the values of the thermal properties for different brain structures and the tumor.

**Table 1:** Thermal properties of human head tissues and tumor

**Tissues**	**ρ**	**C**	**k**	**Q**_**met**_	**B**
	**kg/m**^**3**^	**J/kg.°C**	**W/m.°C**	**W/kg**	**W/m**^**3**^**.°C**
Brain/gray matter	1039	3675	0.57	6.833	35000
Brain/white matter	1043	3621	0.5	6.807	35000
Cerebellum	1040	3640	0.53	6.827	35000
CSF	1007	4191	0.6	0	0
Ear cartilage	1097	3500	0.45	0.2	9100
Eye sclera	1090	3664	0.4	0	0
Fat	916	2524	0.25	0.328	916
Medulla	1039	3675	0.57	6.833	35000
Mouth cavity	1.2	1006	0.03	0	0
Muscles	1041	3546	0.53	0.461	3360
Pons	1039	3675	0.57	6.833	35000
Skin	1100	3437	0.35	1.473	9100
Sinus	1.2	1006	0.03	0	0
Skull	1990	1300	0.39	0.307	1000
Tongue	1041	3546	0.53	0.461	13000
Ventricles	1007	4191	0.6	0	0
Tumor	1043	3621	0.5	6.807	35000

The brain tumor conductivity and dielectric constant were assigned two different values of σ_1_ = 0.595 S/m, ϵ_1_ = 38.836 and σ_2_ = 1.21 S/m, ϵ_2_ = 63.259 respectively. The cancer contrast was 1:1 in the electrical conductivity and relative permittivity compared to the surrounding tissue in the first case. In the second case, the cancer contrast was ~ 2:1 relative to the surrounding tissue in the electrical conductivity and ~ 1.6:1 in the relative permittivity. The 1:1 contrast case was adopted to eliminate any enhancement in the selectivity of microwave absorption in the brain tumor due to the contrast in the electrical properties and was considered a control case to test the focusing ability of the proposed hyperthermia system. This contrast case was tested in the optimized proposed hyperthermia system in the presence of the 3D realistic head model. The second contrast case was simulated based on actual measured electrical properties reported in the literature for brain cancer tissues [29,30]. The contrast in the electrical conductivity also agrees with measured values reported for breast cancer tissues while the contrast in the dielectric permittivity was a little higher (1.6 compared to 1.2) [31]. The thermal properties of the brain tumor were similar to those of the surrounding WM. A convective boundary condition was employed at the skin air interface with a heat transfer coefficient of 8 W/m^2^K [32].

### Antenna models

Two patch antennas with two different substrates: a silicone substrate and a foam substrate assigned dielectric constants of ε_r_ =11.9 (tangent loss tan δ =0.0437) and ε_r_ = 1.002 respectively, were designed, analyzed and optimized for use in the brain hyperthermia applicator. The first antenna design consisted of a conducting ground plane (13.5 × 13.5 cm^2^) and a conducting patch (4.8 × 4.8 cm^2^) at a distance h = 1 mm from the groundplane. The second antenna consisted of a conducting ground plane (60 × 40 cm^2^) and a conducting patch (16 × 8 cm^2^) at a distance h = 4 mm from the groundplane. The conducting patch and the ground plane of each antenna were assigned electrical properties of copper. The polarization of the antenna is along its length.

Input power was set to 500 W and 50 W for the first and second antennas respectively. Simulations were carried out with harmonic excitation signals. The selected resonant frequency used in our computations was 915 MHz since it was found to be within the range of promising authorized frequency candidates for head and neck applicators [33]. The return loss (S_11_) for the two antennas was computed and several optimization steps were performed to fine tune the antenna to resonate at the desired frequency when placed in the hyperthermia applicator system model.

The efficiency of an antenna is mainly dependent on the antenna's return loss *S*_11_ — the ratio of power reflected to power input. *S*_11_ is typically measured on a decibel scale as

(1) $$S_{11}=10\log_{10}\frac{P_{r}}{P_{i}}$$

where *P*_*r*_ is the reflected power and *P*_*i*_ is the input power. Since *S*_11_ is measured on a decibel scale, smaller *S*_11_ indicates greater power coupled to the brain tissue. The 3D radiation patterns of the foam substrate antenna inside the hyperthermia system model with and without the presence of the head model were also computed.

### FDTD analysis

The SEMCAD X version 14 (SPEAG, Zurich, Switzerland), a commercial finite-difference time-domain (FDTD) based program, was used to compute the electromagnetic energy SAR deposited in brain tissues and the tumor as well as the thermal profiles in these tissues. The SAR is defined as the power absorbed into the unit mass of tissue.

(2) $$SA=\frac{\sigma }{2\rho }E_{i}^{2}$$

Where, E_i_ is the peak value of electric field component. The constants σ and ρ denote the conductivity and mass density of the tissue respectively. The computed SAR values were then substituted as the heat source into the Pennes bioheat equation to compute the temperature distribution in different head models as well as in the tumor. The bioheat equation is represented as follows:

(3) $$\rho c\frac{dT}{dt}=\nabla .k\nabla T+Q_{r}+Q_{m}-\rho_{\mathrm{b\iota}}c_{\mathrm{b\iota}}w_{\mathrm{b\iota}}\left(T-T_{\mathrm{b\iota}}\right)$$

where *ρ* is the density of tissue, *c* is the specific heat capacity, *T* is the temperature of tissue, *k* is the thermal conductivity, *Q*_r_ is the regional heat delivered by the source (SAR), Q_m_ is the power generated by metabolism, *ρ*_bl_ is the density of blood, *c*_bl_ is the specific heat capacity of blood, *w*_bl_ is the blood perfusion, and *T*_bl_ is the temperature of blood. B represents the term associated with blood flow and equals *ρ*_bl_*c*_bl_*w*_bl_.

The entire computational domain is divided into voxels, i.e., small cubical elements. SEMCAD provides the possibility of grid refinement since the grid lines do not have to be spaced homogeneously. A maximum of grid stepping between λ/15 and λ/20 is indicated for sufficient accuracy for a variable grid. The spatial discretization of the computational grid was < 1.5 mm. Grid refinements at small brain structures, antenna and half ellipsoidal chamber were used to increase the accuracy of the model while retaining acceptable computational times and satisfying the stability criterion. In our simulations, a twelve-layered UPML was adopted as the absorbing boundary to truncate the computational domain. Convergence was assessed by monitoring the convergence of the S_11_ parameters from iteration to iteration and when the difference in the maximum value of multiple subsequent periods has decreased to a few percent. The temperature increase in the human head was calculated for an exposure time of 30 minutes.

## Numerical results

Various simulations were carried out at 915 MHz to test the applicability of the proposed hyperthermia system model. Two patch antennas having different substrate materials were used to irradiate three different head models and the field distribution inside the proposed chamber, with and without the presence of the human head model was computed. SAR and thermal profiles were also calculated in different brain tissues as well as in the tumor.

The return loss *S*_11_ for both antennas was calculated and their dimensions were optimized to fine tune the antennas to resonate at the desired frequency.

**Figure 3:**
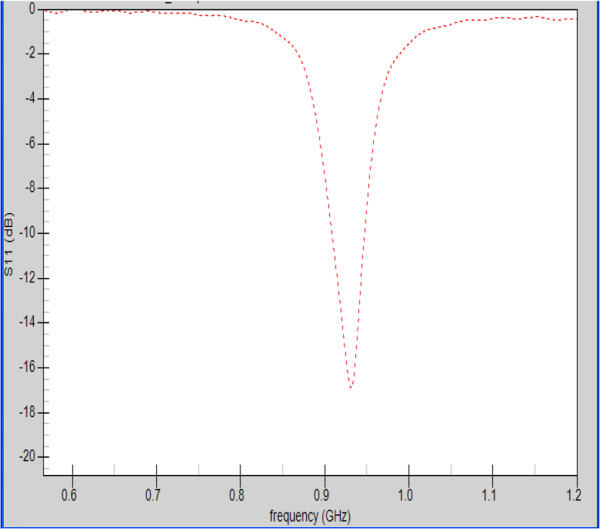
**Return loss S**_**11**_**for the patch antenna with a silicone substrate inside the hyperthermia chamber model.**

Figure 3 shows the return loss for the patch antenna with a silicone substrate when the antenna is placed at one of the focal points of the hyperthermia chamber model. A value of approximately −17 dB is observed for the S_11_ at 915 MHz.

**Figure 4:**
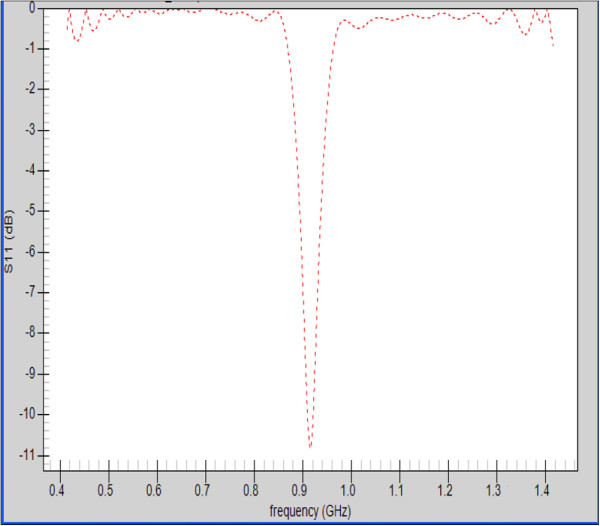
**Return loss S**_**11**_**for the patch antenna with a foam substrate inside the hyperthermia chamber model.**

**Figure 5:**
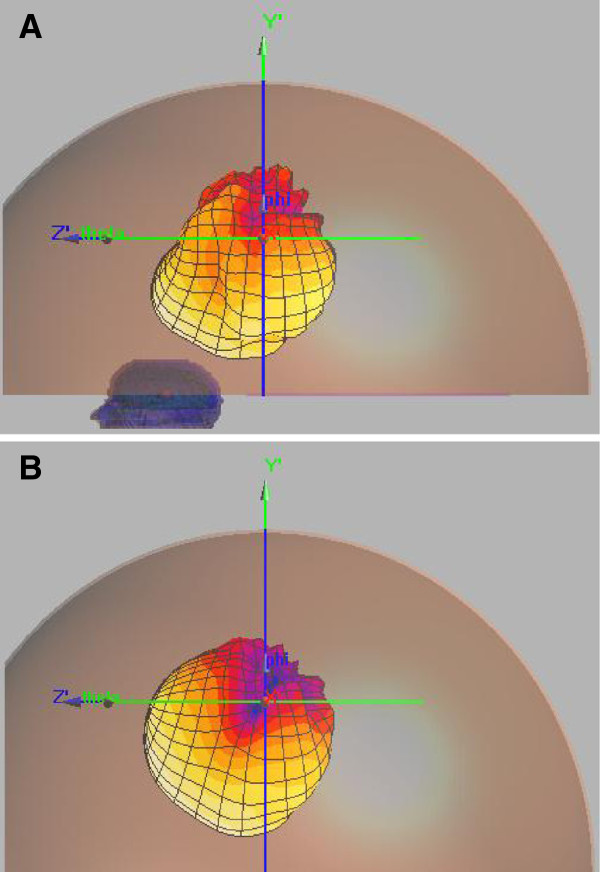
3D radiation patterns of the foam substrate antenna inside the hyperthermia chamber model (A) with the head model present, (B) no head model present.

A return loss S_11_ of −11 dB was observed for the second antenna having a foam substrate when the antenna was placed at one of the foci of the hyperthermia chamber model at the desired frequency (see Figure 4). The 3D radiation patterns of the foam substrate antenna inside the hyperthermia system model with and without the presence of the head model were computed and depicted in Figure 5 A and B at a point inside the proposed hyperthermia chamber. In the direction towards the head, radiation is reduced due to the power absorbed by the head. In the opposite direction there is even a slight increment in radiation, possibly due to partial reflection from the head. A similar radiation pattern was observed for the silicone substrate antenna.

### Focusing properties of the proposed system in the absence of the head model

**Figure 6:**
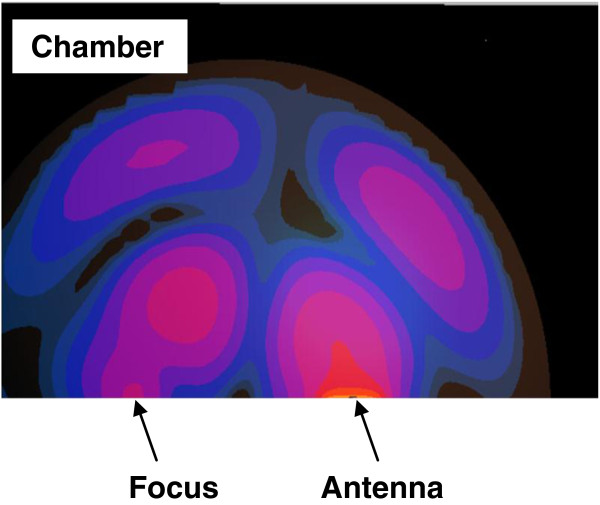
Electric field distribution inside the hyperthermia chamber model at 915 MHz.

The focusing properties of the hyperthermia system model were investigated initially without the presence of the human head model. The electric field distribution inside the proposed system is depicted in Figure 6. It is observed that the radiated energy emitted by the antenna placed at one focus converges on the other focal point. Thus, the geometrical focal point and the electromagnetic convergence area coincide. A 3-dB focusing region of approximately 3 cm is observed in the absence of the head model. Both antenna configurations show a similar electric field pattern inside the hyperthermia system model without the presence of the human head model.

### SAR results

#### SAR in the uniform spherical head model

**Figure 7:**
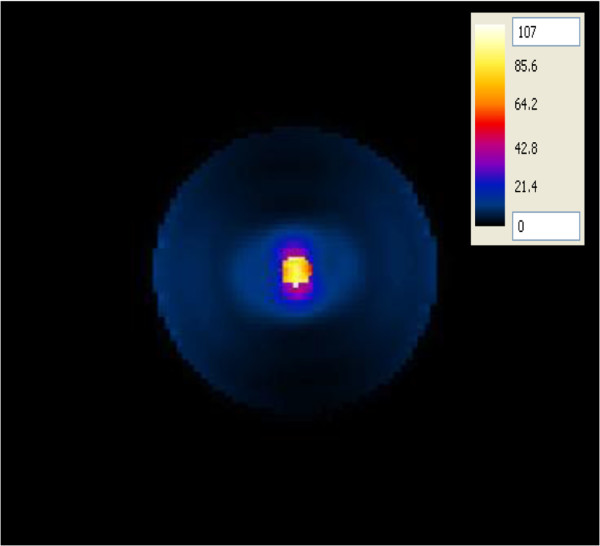
SAR distribution (in mW/g) in an axial plane of the spherical head model when placed at one of the focal points of the hyperthermia chamber model and the antenna with a foam substrate placed at the other focal point.

The SAR was computed in a simple spherical head model composed of skull, average brain tissue and a tumor (Figure 7). The antenna having a foam substrate was used to irradiate this simple head model. In this configuration, the ~ 2:1 contrast between the tumor and the surrounding WM case was adopted. A centrally located region of high SAR (107 mW/g) is observed (yellow color). This high SAR region coincides with the tumor placed at the center of the spherical head model. A similar pattern was observed when using the antenna with a silicone substrate.

#### SAR in the layered concentric spherical head model

**Figure 8:**
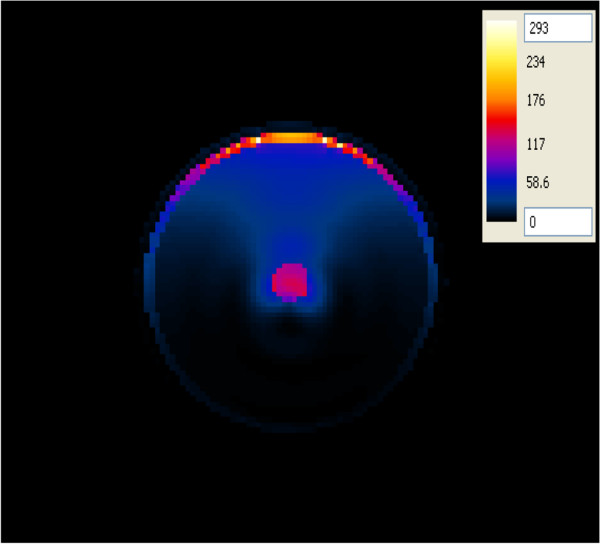
SAR distribution (in mW/g) in an axial plane of the concentric spherical head model when placed at one of the focal points of the hyperthermia chamber model and the antenna with a foam substrate placed at the other focal point.

The SAR was computed in a more complicated head model composed of concentric spheres representing different brain tissues (skull, CSF, GM and a tumor) which was placed at one of the foci of the hyperthermia chamber model (Figure 8). The antenna having a foam substrate was used to irradiate this head model. In this configuration, the ~ 2:1 contrast between the tumor and the surrounding WM case was adopted. The SAR pattern shows a centrally located region of high SAR within the tumor as well as in the outer thin CSF layer. A similar pattern was observed when using the antenna with a silicone substrate. Using more tissues in the head model caused different values of SAR in the tumor (120 mW/g) compared to the simpler head model (107 mW/g). On the other hand, unwanted higher values of SAR, compared to those in the tumor, were observed in the CSF layer (293 mW/g). The presence of the high water content CSF layer in the layered model caused better energy focusing off the chamber walls into the tumor. Thus, indicating the importance of using more complicated head models to account for tissue heterogeneities in the brain.

#### Focusing properties of the Proposed System in the presence of the 3D realistic head model

**Figure 9:**
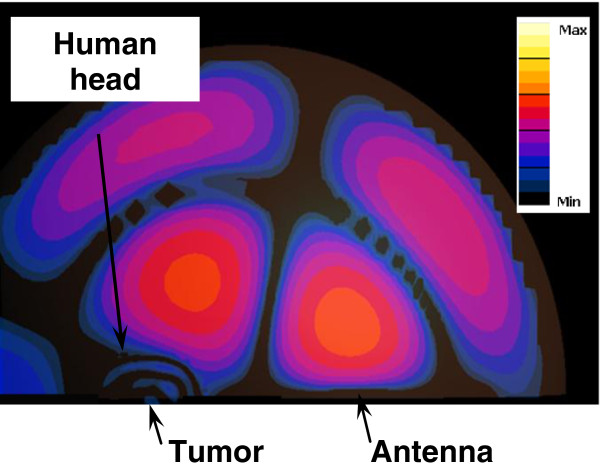
Electric field distribution inside the hyperthermia chamber model in the presence of the 3D head model containing a deep seated tumor at 915 MHz.

The focusing properties of the half ellipsoidal chamber were investigated in the presence of the realistic head model containing the tumor. The electric field distribution inside the proposed hyperthermia system is depicted in Figure 9. The center of the brain tumor was placed at one focal point and either antenna placed at the other focal point. Penetration of the field is observed and focusing on the 2 cm brain tumor which coincides with the focal point is achieved. The radiated energy was mainly localized in the tumor as well as the high water content tissues in the brain (CSF and the ventricles). It is worth noting that similar electric field patterns were observed in the hyperthermia system model with the realistic head model present due to irradiation by either antennas and for the two electrical properties contrast cases adopted in this study (the 1:1 contrast and the ~ 2:1 contrast between the tumor and the surrounding WM).

#### SAR in the realistic 3D head model with a deep seated tumor

**Figure 10:**
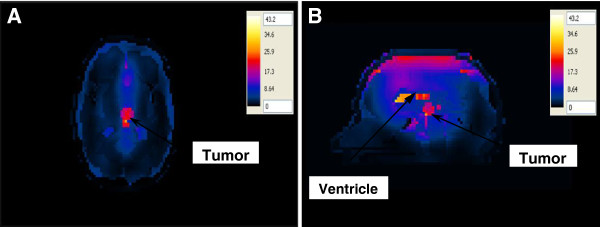
SAR distribution (in mW/g) in the realistic head model with a deep seated tumor placed at one of the focal points of the hyperthermia chamber model and the antenna with a silicone substrate placed at the other focal point for the ~2:1 contrast case between the tumor and the surrounding tissue (A) axial view and (B) sagittal view.

The SAR was computed in the brain tissues when the deep seated tumor in the head was placed at one of the focal points of the hyperthermia chamber model system and the antenna was placed at the other focal point. In the first configuration, the antenna having a silicone substrate was used to irradiate the head (Figure 10A, B). Localization of the SAR was observed at the tumor in the axial and sagittal views, but the maximum SAR was located at the ventricles in the sagittal view. SAR distributions, although small compared to the ventricles, were also located in the outer thin CSF layer of the head. Higher SAR values observed in the ventricles is due to the fact that these brain structures contain CSF. Using more tissues in the head model caused lower values of SAR in the tumor compared to the previous head models.

**Figure 11:**
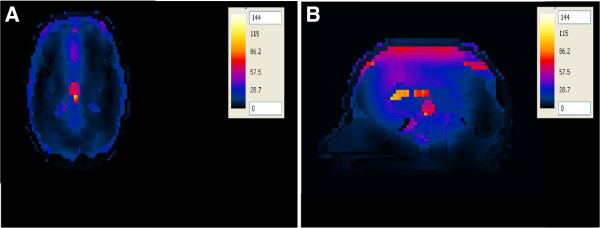
SAR distribution (in mW/g) in the realistic head model with a deep seated tumor placed at one of the focal points of the hyperthermia chamber model and antenna with the foam substrate placed at the other focal point for the 1:1 contrast case between the tumor and the surrounding tissue (control case) (A) axial view and (B) sagittal view.

In the second configuration, the antenna with a foam substrate was placed at the other focal point opposite to the head (Figure 11A, B). In this configuration, the 1:1 contrast between the tumor and the surrounding WM case was simulated. Although the observations on the SAR patterns were similar to those computed for the silicone antenna, much higher SAR values are assessed in brain tissues. Thus, indicating the advantage of using an antenna with a higher efficiency.

**Figure 12:**
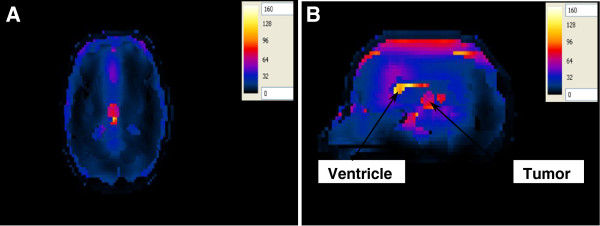
SAR distribution (in mW/g) in the realistic head model with a superficial tumor placed at one of the focal points of the hyperthermia chamber model and antenna with the foam substrate placed at the other focal point for the ~2:1 contrast case between the tumor and the surrounding tissue (A) axial view and (B) sagittal view.

When the ~ 2:1 contrast between the tumor and the surrounding WM case was adopted, a similar SAR pattern was observed in the tumor in both the axial view and the sagittal view (Figure 12A, B). Higher SAR values are observed in the tumor compared to the 1:1 contrast case. Increasing the input power to the foam antenna from 50 W to 64 W caused the SAR value for the 1:1 contrast case in the tumor to increase to the same SAR value observed for the ~2:1 contrast case.

It is also worth noting that the SAR value in the tumor was less than those computed in the two simpler head models due to the complexity of the head geometry and the presence of more brain structures.

#### SAR in the realistic 3D head model with a superficial tumor

**Figure 13:**
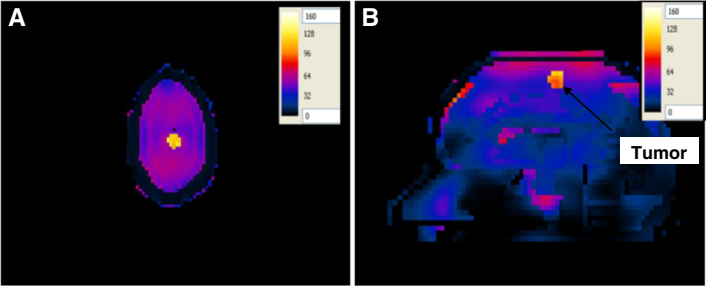
SAR distribution (in mW/g) in the realistic head model with a superficial tumor placed at one of the focal points of the hyperthermia chamber model and antenna with the foam substrate placed at the other focal point for the cancer contrast ~ 2:1 case relative to the surrounding tissue (A) axial view and (B) sagittal view.

The brain tumor was placed at a superficial location inside the realistic 3D head model instead of being deeply seated within the brain. This is analogous to a clinical situation where all the physician needs to do is to place the center of the brain tumor wherever it is located in the head at the other focal point of the hyperthermia system. The SAR was computed in the realistic 3D head model with its superficial tumor placed at one of the focal points of the hyperthermia chamber model and the antenna with a foam substrate placed at the other focal point (Figure 13A, B). In this configuration, the ~ 2:1 contrast between the tumor and the surrounding WM case was adopted. A localized SAR pattern was observed at the superficial tumor in both the axial view and the sagittal view. Smaller values of SAR (approximately 20% of the maximum SAR value) were observed in the outer CSF layer and the ventricles. Similar SAR patterns would be observed when using the silicone substrate antenna with the exception that lower values of SAR would be present in the head.

### Temperature results

#### Temperature in the realistic 3D head model with a deep seated tumor

**Figure 14:**
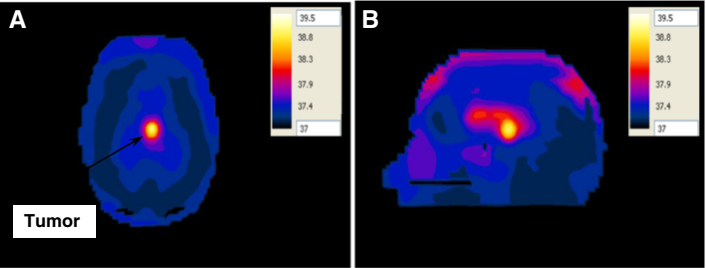
Temperature distribution (in °C) inside the deep seated tumor in the head model placed at one of the focal points of the hyperthermia chamber model and the antenna having a silicone substrate placed at the other focal point for the ~2:1 contrast case between the tumor and the surrounding tissue (A) axial view and (B) sagittal view.

It’s very important to calculate the temperature due to hyperthermia therapy process, because the SAR values alone are not sufficient to assess the effectiveness of the hyperthermia therapy process. The SAR values are substituted into the bioheat equation to compute the temperature in different brain tissues. The temperature values were calculated when the brain tumor within the head was placed at one of the focal points of the hyperthermia chamber model system and the antenna placed at the other focal point. In the first configuration, the antenna having a silicone substrate was used to irradiate the head (Figure 14A, B). The ~ 2:1 contrast between the tumor and the surrounding WM case was adopted. A localized temperature of 39.5°C was observed in the tumor. Another observation worth noting is that the temperature distribution does not coincide exactly with the SAR distribution in brain tissues since the highest temperature exists in the tumor in the sagittal plane while the temperature in the outer CSF layer and ventricles was less than 38°C (see Figure 10B, Figure 14B).

**Figure 15:**
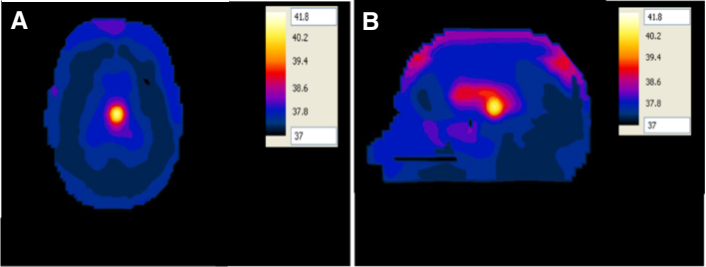
Temperature distribution (in °C) inside the deep seated tumor in the head model placed at one of the focal points of the hyperthermia chamber model and the antenna having a foam substrate placed at the other focal point for the 1:1 contrast case between the tumor and the surrounding tissue (control case) (A) axial view and (B) sagittal view.

In the second configuration, the antenna with a foam substrate was placed at the other focal point opposite to the head (Figure 15A, B). In this configuration, the 1:1 contrast between the tumor and the surrounding WM case was adopted. A temperature of 41.8°C was observed in the deep seated tumor while the temperature of the surrounding healthy brain tissue was below 37.8°C. Increasing the input power to the antenna from 50 W to 64 W caused the temperature in the tumor to increase to therapeutic values of 43.4°C while keeping the temperature of the surrounding healthy brain tissue below 38.5°C.

**Figure 16:**
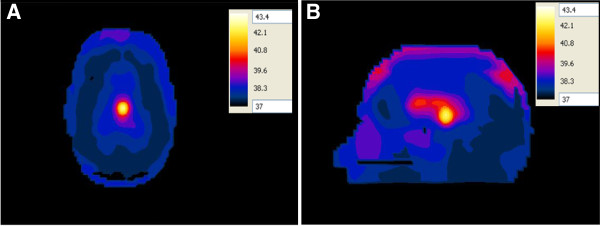
Temperature distribution (in °C) inside the deep seated tumor in the head model placed at the one of the focal points of the hyperthermia chamber model and the antenna having a foam substrate placed at the other focal point for the ~2:1 contrast case between the tumor and the surrounding tissue (A) axial view and (B) sagittal view.

When the ~ 2:1 contrast between the tumor and the surrounding WM case was adopted, a localized temperature of 43.4°C was observed in the tumor while the temperature in the outer CSF layer and ventricles was less than 39°C (Figure 16A, B).

Again, another observation worth noting is that the temperature distribution does not coincide with the SAR distribution in brain tissues since the highest temperature exists in the tumor in the sagittal plane (see Figure 12B, Figure 16B). In addition, higher temperatures are observed in the tumor compared to those computed when the head was irradiated by an antenna with a silicone substrate placed in the hyperthermia chamber model (Figure 14).

#### Temperature in the realistic 3D head model with a superficial tumor

**Figure 17:**
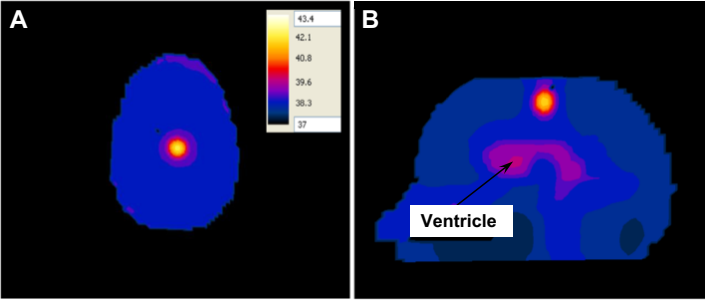
Temperature distribution (in °C) inside the superficial brain tumor in the head model placed at one of the focal points of the hyperthermia chamber model and the antenna having a foam substrate placed at the other focal point for the ~2:1 contrast case between the tumor and the surrounding tissue (A) axial view and (B) sagittal view.

The temperature values were computed in the realistic 3D head model with its tumor placed at one of the focal points of the hyperthermia chamber model and the antenna with a foam substrate placed at the other focal point (Figure 17A, B). In this configuration, the brain tumor was placed at a superficial location inside the realistic 3D head model instead of being deeply seated within the brain. The ~ 2:1 contrast between the tumor and the surrounding WM case was adopted. A localized temperature of 43.4°C was observed in the tumor while the temperature in the ventricles was less than 38.8°C.

## Discussion

The focus of this study was to numerically design a non invasive hyperthermia applicator system capable of adequately heating deep seated as well as superficial brain tumors embedded in head models using the finite difference time domain method.

The proposed hyperthermia system design is shown to be capable of effectively heating deep seated as well as superficial brain tumors using inexpensive, simple, and easy to fabricate constituents. To test the effectiveness of hyperthermia treatment, the specific absorption rate (SAR) deposited in brain tissues must be high enough to produce sufficient temperature rise in brain tumors. The hyperthermia chamber system should be capable of rising the temperature of brain tumors to values above 42°C without harming surrounding healthy tissues. Therefore, SAR patterns and temperature distribution were computed and compared using two different antenna designs to irradiate three different head models.

Two improvement strategies were performed on all of the hyperthermia system configurations to adequately ensure sufficient and focused energy deposition (SAR) and temperature distribution in brain tumors. In the first approach, the head models varied from a very simple spherical head model to a layered concentric spherical model and finally to a more complicated and realistic 3D head model. Each of the head models contained a tumor. SAR distributions in the spherical head models, using any of the two patch antenna configurations, were observed to be different than those computed in the realistic 3D head model. A localized SAR pattern was observed in the tumor placed at the center of the simple spherical head model (see Figure 7). Karanasiou et al., [25] used a similar simple head model in their study, but several hot spots were observed in their head model. This may be attributed to the antenna they have used or to their applicator design needing further optimizations. When using the layered spherical head model, a centrally located region of high SAR within the tumor as well as an undesired superficial region (coinciding with the outer CSF layer) of the head was observed (see Figure 8). The CSF is a high water content compartment which has a relatively high dielectric constant and conductivity compared to the surrounding WM tissue. Most of the energy was absorbed in the CSF layer compared to the deep seated tumor located centrally within the head. This observation is consistent with those used to assess SAR values deposited in the head due to the exposure to microwave frequencies emanating from cellular phones [34] and MRI equipment [35]. SAR patterns computed in the realistic 3D head model using any of the two antenna configurations showed a distorted SAR distribution and hot spots in the ventricles as well as in the brain tumor compared to those observed in the simple head models. The two simpler head models overestimated the values of SAR deposited in the tumor. Thus, highlighting the significance of using a 3D realistic head model with many tissue heterogeneities to assess the effectiveness of any hyperthermia system design.

The second approach involved using two different patch antennas of optimized dimensions in the hyperthermia setup to enforce radiated waves to propagate and reflect from the upper walls of the chamber towards the target region (tumor) only.

The antenna design varied from an antenna with a silicone substrate configuration to an antenna design with a foam substrate. Two parameters of the antenna are affected by the substrate material, the size of the antenna and its efficiency [36]. High dielectric substrate materials such as the silicone antenna can reduce the antenna size at the expense of the antenna efficiency. Thus, there is trade-off between the size of the antenna and its efficiency. The efficiency of the antenna was assessed by its capability to sufficiently cause localized heating of the brain tumor using much less input power to the antenna. Although the silicone antenna had a smaller size compared to the foam antenna, it was fed with a much higher input power of 500 W. This antenna design caused a temperature rise of 39.5°C in the tumor which wasn't high enough to reach therapeutic levels. Experimental data has shown that the threshold temperature increase of 3.5°C has been noted to be an allowable limit in the brain which does not lead to physiological damage [37]. The silicone antenna design also had a low radiation efficiency due to high losses in the silicone substrate (loss tangent tan δ = 0.0437). The radiation efficiency of the microstrip antenna is defined as the ratio between the power radiated into space and the total input power which is dependent on the power dissipated by the substrate dielectric loss.

When a superficial tumor located in the head model was placed at the focal point of the chamber and irradiated by the foam substrate antenna, the proposed air filled hyperthermia chamber model was capable of heating the brain tumor using a single frequency (Figure 17A, B). Whole ellipsoidal chambers employed in another study [26] to deposit energy into brain regions using different frequencies and dielectric matching layers may cause patients discomfort due to psychological factors compared to the hyperthermia chamber system proposed in this study.

Our results also revealed that the SAR patterns in brain tissues are not correlated in a simple manner to the temperature distribution in these tissues. Comparing Figures 10, 11, 12 and 13 with Figures 14, 15, 16 and 17, we find that the temperature increase distribution does not coincide exactly with the SAR pattern. In particular, although several hot spots are observed in the SAR patterns, localized and smoother patterns are observed in the temperature distribution. These results are consistent with those of previous studies [38,39]. The SAR is known to be not very smooth due to the variation in the electrical conductivity between different brain tissues [40,41] while the temperature rise distribution is rather smooth due to thermal transfer mechanisms between brain tissues.

The proposed system is designed to achieve focused power deposition in the tumor through constructive reflections off the upper chamber walls, but selective absorption of the electromagnetic waves takes place and causes power deposition in the ventricles as well. There is not good spatial correlation between SAR and temperature, i.e., the peak temperature in certain brain tissues is not directly related to the peak SAR value and location. Similar observations were reported in other studies due to the exposure of the human head to microwave frequencies [37,38,42]. This mismatch is attributed to the complex, multifactorial relationship between temperature and SAR. The peak temperature not only depends on tissue properties such as thermal conduction, metabolic heat generation, the complex heterogeneous geometry of the brain structures, but also largely depends on the wide variance in blood perfusion rates of different tissues. The complex shape of the ventricles may also lead to a greater thermal diffusion surface area compared to the simple spherical tumor causing further reduction in the temperature.

The proposed hyperthermia treatment system was shown to be effective in heating the target tumor while keeping the healthy brain tissue way below the thermal damage limit. Although hot spots were observed in the ventricles, these temperatures (< 39°C) were below those observed in another study using a time-multiplexed beamforming technique (< 41°C) [27]. The circulation of the CSF in the CSF layer and the ventricles were not accounted for in our model which may reduce the temperatures observed in these structures due to the cooling effect arising from circulation. The turnover of the entire volume of CSF is 3–4 times per day [43]. Thus, the temperature in the ventricles is considered the upper limit of temperature increase. Since hyperthermia is used alone or as an adjuvant to other treatment modalities, the physician must decide whether to decrease the input power to the antenna (decrease the temperature in the tumor and the ventricles) and prolong the treatment for more than 30 minutes or to take the risk of treatment for a particular patient.

The focusing performance of the proposed hyperthermia system was tested by assigning different electrical properties to the tumor compared to the surrounding tissue due to uncertainties and the lack of precise values of these properties in the literature. Several studies have shown that malignant tissue has a higher electrical conductivity and permittivity than normal tissue in the breast and the human liver at microwave frequencies [31,44,45]. The difference between healthy tissue and tumor is attributed to the increased water content of the latter, which results in an increased permittivity and an increased conductivity [46].

The proposed hyperthermia system caused localized SAR in the tumor for both cases of contrast between the tumor and the surrounding WM. Although the temperature rise in the tumor was less for the 1:1 contrast case as compared to the temperature rise for the ~ 2:1 contrast case (Figure 15 compared to Figure 16), increasing the input power to the antenna compensated for the observed difference. Thus, indicating the capability of the proposed system to cause heating of the brain tumor to therapeutic values despite the contrast in the electrical properties.

It is also worth noting that, calculating the temperature increase with the Pennes bioheat equation may have some limitations. In particular, the effect of vasculature which causes cooling of surrounding tissues was excluded in our study. However, in order to raise the temperature of the tumor to higher values using the proposed hyperthermia applicator, we only need to increase the input source power to the antenna which will result in higher SAR values and higher temperatures in the brain tumor.

We have built the hyperthermia chamber system and the antenna with a foam substrate using simple inexpensive materials. Preliminary proof of concept was achieved by placing another antenna at the location of the brain tumor and measuring the S_21_. In future work, experimental SAR verification as well as temperature measurements in different tissues of a phantom head model would be conducted to test the effectiveness of the proposed hyperthermia chamber model.

## Conclusions

We utilized the FDTD method to design, model and simulate a low cost and easy to fabricate noninvasive air filled hyperthermia applicator system capable of heating deep seated as well as superficial brain tumors. Accurate modeling of the head geometry (realistic 3D head model) is of vital importance since simple models do not correctly predict SAR patterns. Careful design and selection of the antenna as well as the partial half ellipsoidal chamber proved suitable for raising the temperature to sufficient therapeutic values in the target tumor within the brain. Our results may form the basis for a clinical prototype. The operator of the proposed hyperthermia system only needs to place the center of the brain tumor at a pre-specified location (one of the foci) and excite the antenna at a single frequency of 915 MHz.

## Competing interests

The authors declare that they have no competing interests.

## Authors’ contributions

All authors contributed equally to this work. All authors read and approved the final manuscript.
